# Hypotonic Stress Induces Fast, Reversible Degradation of the Vimentin Cytoskeleton via Intracellular Calcium Release

**DOI:** 10.1002/advs.201900865

**Published:** 2019-07-22

**Authors:** Leiting Pan, Ping Zhang, Fen Hu, Rui Yan, Manni He, Wan Li, Jingjun Xu, Ke Xu

**Affiliations:** ^1^ The Key Laboratory of Weak‐Light Nonlinear Photonics Ministry of Education School of Physics and TEDA Institute of Applied Physics Nankai University Tianjin 300071 China; ^2^ Department of Chemistry University of California Berkeley CA 94720 USA; ^3^ Chan Zuckerberg Biohub San Francisco CA 94158 USA

**Keywords:** calpains, cytoskeletal ultrastructure, hypotonic stress, intracellular calcium release, super‐resolution microscopy, vimentin intermediate filaments

## Abstract

The dynamic response of the cell to osmotic changes is critical to its physiology and is widely exploited for cell manipulation. Here, using three‐dimensional stochastic optical reconstruction microscopy (3D‐STORM), a super‐resolution technique, the hypotonic stress‐induced ultrastructural changes of the cytoskeleton of a common fibroblast cell type are examined. Unexpectedly, these efforts lead to the discovery of a fast, yet reversible dissolution of the vimentin intermediate filament system that precedes ultrastructural changes of the supposedly more dynamic actin and tubulin cytoskeletal systems as well as changes in cell morphology. In combination with calcium imaging and biochemical analysis, it is shown that the vimentin‐specific fast cytoskeletal degradation under hypotonic stress is due to proteolysis by the calcium‐dependent protease calpain. The process is found to be activated by the hypotonic stress‐induced calcium release from intracellular stores, and is therefore efficiently suppressed by inhibiting any part of the IP_3_‐Ca^2+^‐calpain pathway established in this study. Together, these findings highlight an unexpected, fast degradation mechanism for the vimentin cytoskeleton in response to external stimuli, and point to the significant, yet previously overlooked physiological impacts of hypotonic stress‐induced intracellular calcium release on cell ultrastructure and function.

## 1 Introduction

The intricate inner machinery of the cell depends critically on water homeostasis to regulate intracellular concentrations. Differences in intracellular and external concentrations (osmotic pressures) give rise to osmosis‐induced cell volume and behavior changes, which constitute essential processes in physiology and pathology.^1^, ^2^, ^3^ As a cell manipulation tool, hypo‐osmotic swelling has been widely utilized for intracellular delivery^4^, ^5^, ^6^ and the modulation of membrane tension.^7^, ^8^, ^9^ Recent work further established osmotic volume change as a key method to control intracellular protein concentration for studies on protein crowding and protein–protein interactions.^10^, ^11^ Consequently, it is of both fundamental and practical importance to understand if osmotic effects would lead to significant intracellular structural changes, and if so, how fast do such changes occur, whether they are reversible and/or suppressible as well as what mechanisms drive such processes. The cytoskeleton plays essential roles in the cell volume regulation under osmotic stress,^12^ a natural consequence given the pivotal role of the cytoskeleton in cell structure and function.^13^, ^14^, ^15^

In particular, the intermediate filament system critically defines the mechanical properties of vertebrate cells.^14^, ^16^ Vimentin is a major component of the intermediate filament system for most cells in culture;^17^ besides supporting the cell shape, it also anchors organelles and impedes intracellular movement by doubling the cytoplasmic shear modulus.^14^, ^16^, ^18^ Recent work underscores the dynamic interplays between the vimentin system and the actin and tubulin cytoskeletal systems.^19^, ^20^, ^21^, ^22^ One emerging theme has been that the vimentin system follows the initial structural cues of the more dynamic actin and tubulin systems, but subsequently helps provide structural persistency through its superior stability and rigidity. Unalterable high stability, however, could pose significant challenges when the cell needs to respond quickly to external stimuli, e.g., that due to osmotic stress.

Using three‐dimensional stochastic optical reconstruction microscopy (3D‐STORM) super‐resolution microscopy (SRM),^23^, ^24^ here we examine the hypotonic stress‐induced ultrastructural changes of the cytoskeleton of a common fibroblast cell type at high spatial resolution. Interestingly, among the three major cytoskeletal systems, we find a quick dissolution of the vimentin intermediate filament system, whereas the actin and tubulin systems are mostly unaffected. We next find the structural changes rapidly recover under normal culturing conditions, with a recovering speed notably faster than that of the cell morphology. In combination with calcium imaging and biochemical analysis, we further establish that the rapid, vimentin‐specific cytoskeletal degradation is driven by the calcium binding‐mediated activation of the protease calpain through hypotonic stress‐induced intracellular calcium release, and so is effectively blocked by corresponding inhibitors and calcium chelators. Together, our findings highlight an unexpected fast degradation mechanism for the vimentin cytoskeleton, and point to the significant, yet previously overlooked physiological impacts of hypotonic stress‐induced intracellular calcium release on cell ultrastructure.

## 2 Results

COS‐7 cells were cultured in Dulbecco's modified Eagle medium (DMEM) supplemented with 10% fetal bovine serum (FBS) following standard tissue‐culture protocols. Differential interference contrast (DIC) microscopy (Figure S1, Supporting Information) indicated that after the cells were challenged with the hypotonic stress due to a 50% HBSS (Hank's balanced salt solution) buffer (osmotic pressure: 145 mOsm kg^−1^), no significant changes to cell general morphology was observed at 5 min, although increased membrane ruffling was often noticed at cell edges (Figure S1A, Supporting Information). Meanwhile, cells treated with pure water quickly rounded up within the same time window (Figure S1B, Supporting Information).

**Figure 1:**
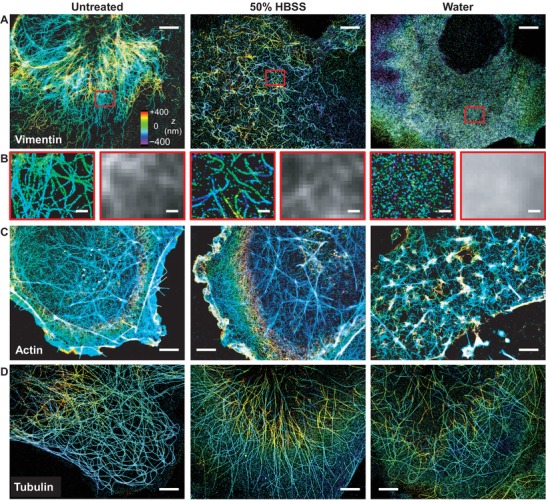
3D‐STORM reveals that hypotonic stress leads to fast degradation of the vimentin, but not the actin and tubulin cytoskeletal systems. A) 3D‐STORM images of immunolabeled vimentin for fixed COS‐7 cells that were untreated (left), treated by 50% HBSS for 5 min (center), and treated by pure water for 5 min (right). Color represents height *z* (color bar: violet denotes closest to the substrate and red denotes farthest away). B) Enlargements of the red boxes in (A), compared to the diffraction‐limited epifluorescence images of the same areas. C) 3D‐STORM images of phalloidin‐labeled F‐actin in fixed COS‐7 cells that were untreated (left), treated by 50% HBSS for 5 min (center), and treated by pure water for 5 min (right). D) 3D‐STORM images of immunolabeled alpha‐tubulin in fixed COS‐7 cells that were untreated (left), treated by 50% HBSS for 5 min (center), and treated by pure water for 5 min (right). Scale bars: A,C,D) 4 µm and B) 500 nm.

To investigate cytoskeletal changes at the ultrastructural level, we fixed the cells for immunofluorescence labeling, and performed 3D‐STORM SRM to achieve ≈20 nm spatial resolution optically.^23^, ^24^, ^25^, ^26^, ^27^ Notably, 3D‐STORM revealed substantial ultrastructural changes in the vimentin intermediate filaments (**Figure** **1**A,B and Figure S2A, Supporting Information), whereas conventional, diffraction‐limited microscopy did not well resolve the filaments (Figure 1B). For the milder treatment of 50% HBSS buffer, where changes in cell morphology were moderate (Figure S1A, Supporting Information), 3D‐STORM showed that the initially long and continuous vimentin filaments, which often ran through the entire cell lengths, all broke down into short segments a few micrometers in length (Figure 1A,B). As a result, the structural network formed by the vimentin filaments (Figure 1A) disintegrated, presumably negating the force‐bearing function of this system.^14^, ^16^, ^18^ More dramatically, cells treated with pure water for the same amount of time were characterized by completely dissolved vimentin (Figure 1A,B). Live‐cell microscopy of cells expressing a fluorescent protein‐tagged version of vimentin further showed that upon water treatment, the integrity of the vimentin cytoskeleton was quickly disrupted within ≈180 s (Figure S3 and Movie S1, Supporting Information).

In comparison, the actin and tubulin cytoskeletal systems were both found to be much less affected by hypotonic stress (Figure 1C,D and Figure S2B,C, Supporting Information). The 50% HBSS treatment did not significantly alter the phalloidin‐labeled cortical actin network (Figure 1C and Figure S2B, Supporting Information; see also more STORM examples of the actin cytoskeleton in our previous work^28^), except that ruffle‐like actin structures were more often observed, in agreement with our DIC results (Figure S1A, Supporting Information). More disrupted cortical actin networks were found for cells treated with pure water (Figure 1C); however, this disruption appeared not to be due to disassembly of the actin filaments, but instead, was consistent with the reorganization of filaments in response to the high membrane tension due to hypotonic swelling.^8^ Meanwhile, the ultrastructure of microtubules was largely unaffected by hypotonic stress (Figure 1D): even for the pure water treatment, microtubules appeared intact, although a somewhat increased staining background was noted, suggesting higher levels of tubulin monomers that did not integrate into the microtubules. Figure S2 in the Supporting Information provides additional examples of the contrasting ultrastructural changes of the three cytoskeletal systems.

**Figure 2:**
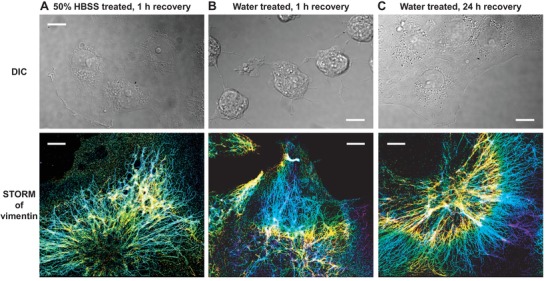
The degraded vimentin network quickly recovers. A) DIC microscopy (top) and 3D‐STORM of vimentin (bottom) for COS‐7 cells that had been treated with 50% HBSS for 5 min and then allowed to recover in the regular culture medium for 1 h. The same color scale as Figure 1 is used to represent the height *z*. B) DIC (top) and 3D‐STORM of vimentin (bottom) for COS‐7 cells that had been treated with pure water for 5 min and then allowed to recover in the regular culture medium for 1 h. C) DIC (top) and 3D‐STORM of vimentin (bottom) for COS‐7 cells that had been treated with pure water for 5 min and then allowed to recover in the regular culture medium for 24 h. Scale bars: 20 µm (top panels) and 4 µm (bottom panels).

We next investigated if the above ultrastructural changes induced by hypotonic stress were reversible. For cells treated with 50% HBSS, 1 h regrowth in the regular culture medium led to a good recovery of the vimentin cytoskeleton (**Figure** **2**A), so that the reassembled filaments again ran through entire cell lengths, similar to untreated cells. Cell overall morphology also recovered (Figure 2A). Interestingly, for cells that had been treated with pure water, 1 h regrowth in the culture medium also led to the full reassembly of long vimentin filaments, even though the cell morphology was even more abnormal than that was observed right after the 5 min water treatment (Figure 2B). A 24 h regrowth in the culture medium gave satisfactory recovery in terms of both cell morphology and vimentin ultrastructure (Figure 2C). The actin and tubulin cytoskeletal systems, which were less disrupted by the hypotonic stress in the first place, also recovered after regrowth in the regular culture medium (Figure S4, Supporting Information). Together, our results indicate quick breakdown and recovery of the vimentin cytoskeleton under hypotonic stress and regrowth conditions, respectively, and the dynamics of both processes appeared to be substantially faster than that of the cell shape.

The striking contrast we observed for how the different cytoskeletal systems responded to the hypotonic stress prompted us to investigate the underlying mechanisms. Paradoxically, whereas actin filaments and microtubules and both known to be structurally highly dynamic and thus prone to dissociation into monomers,^29^, ^30^ vimentin filaments are often considered highly stable.^17^ Indeed, for structural preservation through chemical fixation, it is well recognized that the proper fixation of actin and microtubules both require strong fixatives like glutaraldehyde and/or specialized cytoskeleton‐stabilizing buffers,^28^, ^31^, ^32^ whereas vimentin filaments are readily preserved with common, less potent fixatives as paraformaldehyde (data not shown). Consequently, the observed fast degradation of vimentin filaments, but not actin filaments or microtubules, under hypotonic stress cannot be explained through the intrinsic stabilities of the different filaments. A recent study proposes that microtubule‐based transport may mediate the reorganization of the vimentin cytoskeleton under hypotonic stress.^33^ We found that in our case, the fast degradation of the vimentin cytoskeleton persisted upon removal of the microtubule cytoskeletal system (Figure S5, Supporting Information).

**Figure 3:**
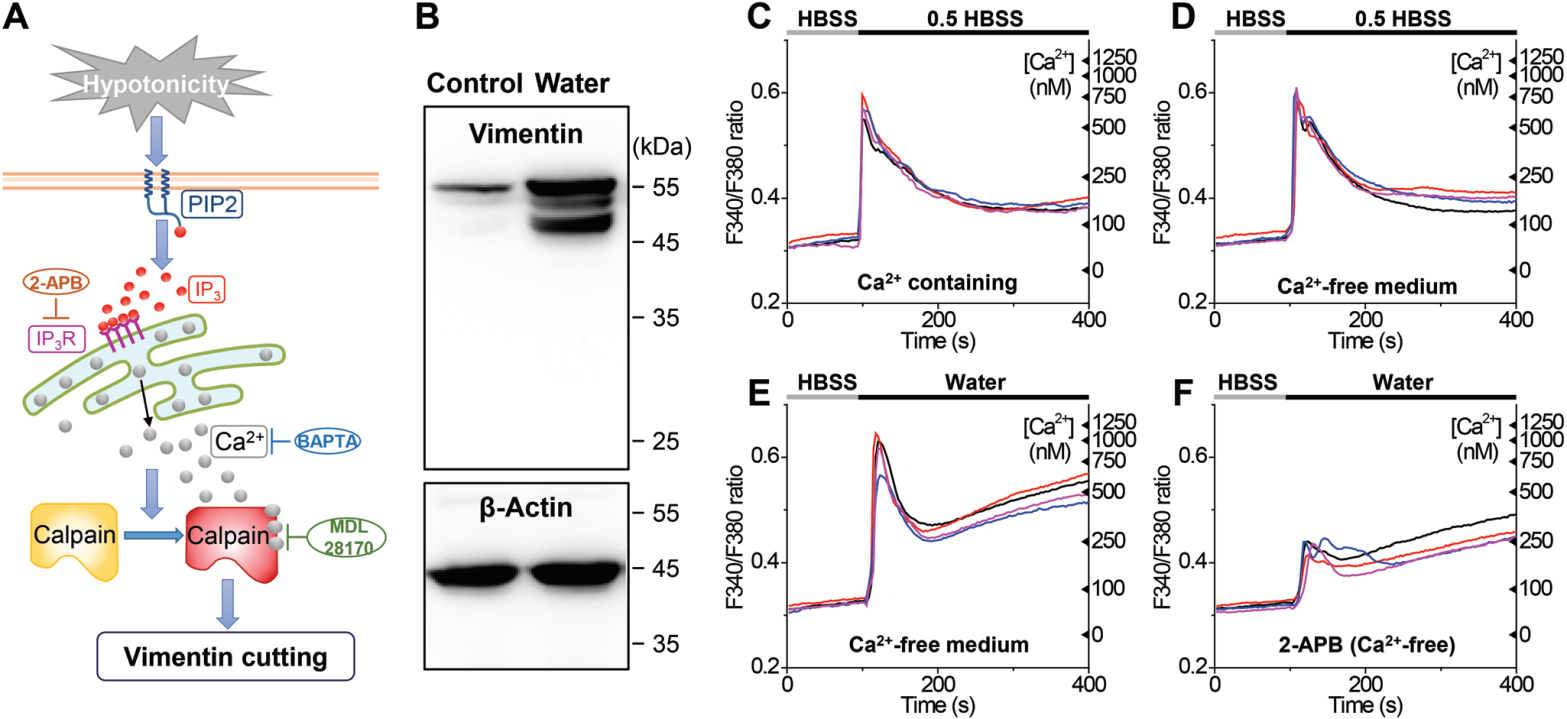
Hypotonic stress induces vimentin cleavage through calcium release from intracellular stores. A) Schematic for the proposed mechanism of fast vimentin degradation under hypotonic stress. Hypotonic stress leads to an increased level of IP_3_ by hydrolysis of phosphatidylinositol 4,5‐bisphosphate (PIP2), which results in calcium release from the ER through the IP_3_ receptor (IP_3_R). Increased cytosolic calcium activates calpain for vimentin cutting. 2‐APB is an antagonist for the IP_3_R, BAPTA is a calcium chelator, and MDL 28170 is a calpain inhibitor. B) Immunoblot analysis of lysates of untreated (control) cells versus cells that had been treated with water for 5 min. C–F) Fluorescence readout of cytosolic Ca^2+^, shown as the fura‐2 fluorescence intensity ratio at 340 nm versus 380 nm excitation (left axis) and the converted Ca^2+^ concentration in nm (right axis), for C) changing the medium from a regular HBSS buffer to 50% HBSS, D) changing the medium from a calcium‐free HBSS buffer to a calcium‐free 50% HBSS buffer, E) changing the medium from a calcium‐free HBSS buffer to pure water, and (F) is the same as (D) but in the presence of 75 × 10^−6^ m of 2‐APB. For each condition, four typical curves are shown from four different cells.

An important degradation pathway of vimentin is the calcium (Ca^2+^)‐dependent proteolysis by calpain family proteins.^34^, ^35^ It has been reported that hypotonic stress leads to increased intracellular calcium concentration;^3^, ^36^ since the calcium‐activation of calpain leads to rapid degradation of vimentin but very slow or no degradation of actin and tubulin,^34^, ^35^ a potential differentiation mechanism may thus be established (**Figure** **3**A).

To test this hypothesis, we performed immunoblot analysis for the lysates of untreated cells and cells that had been subjected to hypotonic stress (Figure 3B and Figure S6, Supporting Information). A single major band of ≈57 kDa was observed for vimentin for the control cells. In contrast, cells that had been treated by water showed two pronounced new bands of lower weights, and intriguingly, all three bands were substantially stronger than the single band observed for the untreated cells. The new low‐weight bands indicate cleavage products, and the weights we observed (≈48 and ≈53 kDa) were consistent with calpain‐mediated vimentin cleavage.^37^, ^38^, ^39^ Notably, for the water‐treated cells, the strength of the cleaved ≈48 kDa band became comparable to the original ≈57 kDa band, indicating that a significant fraction of vimentin was cleaved, as opposed to the control sample, in which the signal of the cleaved bands was significantly lower than the uncleaved band (Figure 3B and Figure S6, Supporting Information). The significantly enhanced blotting signal for all bands in the water‐treated cells further suggests that the proteolysis, and hence cytoskeletal breakdown process, was accompanied by the dissociation of vimentin monomers into the cytosol, which enhanced their detection as we collected the lysate for immunoblot. In comparison, for both samples, β‐actin showed a single band of comparable strengths at ≈45 kDa (Figure 3B). Together, our immunoblot analysis suggests that upon hypotonic stress, calpain cleaves vimentin but not actin.

To further examine the possibility of calcium‐induced activation of calpain, we next measured cytosolic Ca^2+^ concentrations under hypotonic stress using the ratiometric Ca^2+^ indicator fura‐2‐acetoxymethyl ester (fura‐2/AM). Indeed, challenging the cells with 50% HBSS led to a sudden rise in Ca^2+^ signal (Figure 3C). To identify whether this rise was due to an influx from the extracellular medium, we repeated the experiment with calcium‐free HBSS (Figure 3D). Comparable rises in Ca^2+^ signal were noted, thus suggesting that the increase in cytosolic Ca^2+^ level was mostly due to calcium release from intracellular stores. In a similar vein, treating the cells with pure water also led to increased cytosolic Ca^2+^ (Figure 3E). Notably, converting the measured signals to actual Ca^2+^ concentrations indicated levels of >600 × 10^−9^ m and >1 × 10^−6^ m for the 50% HBSS and pure water treatments, respectively (right axes of Figure 3C–E; see also Figure S7, Supporting Information, for a linear scale of Ca^2+^ concentration). In comparison, previous work has shown that inside the cell, calpains are activated for Ca^2+^ concentrations >≈400 × 10^−9^ m.^40^, ^41^

To further probe the mechanism of this calcium release, we found (Figure 3F) that the Ca^2+^ increase was suppressed by the application of 2‐aminoethoxydiphenyl borate (2‐APB), an antagonist for the inositol 1,4,5‐trisphosphate (IP_3_) receptor,^42^ thus suggesting that the calcium release was due to the IP_3_ signaling pathway as opposed to passive leakage from the endoplasmic reticulum (ER). Indeed, it has been suggested that hypo‐osmotic swelling may result in the hydrolysis of phosphoinositides and an increased intracellular level of IP_3_.^3^, ^43^, ^44^

**Figure 4:**
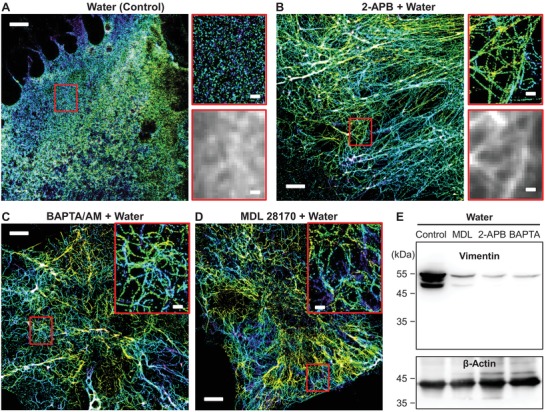
Inhibition of calpain activity or intracellular calcium release preserves the integrity of the vimentin cytoskeleton against hypotonic stress. A) 3D‐STORM image of the immunolabeled vimentin in a control COS‐7 cell sample after treatment with pure water for 5 min, together with zoom‐in of the red box and the corresponding, diffraction‐limited epifluorescence image. The same color scale as Figure 1 is used to represent the height *z*. B) 3D‐STORM image of a sample treated with water for 5 min in the presence of 75 × 10^−6^ m 2‐APB, together with zoom‐in of the red box and corresponding epifluorescence image. C) A sample treated with water for 5 min in the presence of 30 × 10^−6^ m BAPTA/AM. Inset: zoom‐in of the red box. D) A sample treated with water for 5 min in the presence of 30 × 10^−6^ m MDL 28170. Inset: zoom‐in of the red box. Scale bars: 4 µm (main figures) and 500 nm (zoom‐ins). E) Immunoblot analysis of cell lysates corresponding to the conditions in (A)–(D).

To elucidate whether the above‐identified rise in intracellular Ca^2+^ level was indeed the driving force of vimentin degradation, we examined if vimentin degradation could be suppressed by different chemicals that separately block different parts of our proposed IP_3_‐Ca^2+^‐calpain pathway (Figure 3A). Indeed, the inclusion of 2‐APB, which inhibited intracellular calcium release (Figure 3F), substantially suppressed the degradation of vimentin with pure water: 3D‐STORM showed well‐preserved long vimentin filaments through the entire cell lengths (**Figure** **4**B and Figure S8A, Supporting Information), in stark contrast to the fully dissolved vimentin in control samples treated with water alone (Figure 4A). Similar suppression of vimentin degradation was found with the application of *N*,*N*′‐[1,2‐ethanediylbis(oxy‐2,1‐phenylene)]bis[*N*‐[2‐[(acetyloxy)methoxy]‐2‐oxoethyl]]‐, bis[(acetyloxy)methyl] ester (BAPTA/AM), a cell‐permeable calcium chelator (Figure 4C and Figure S8B, Supporting Information). Finally, MDL 28170, a common calpain inhibitor,^45^ also effectively suppressed vimentin degradation (Figure 4D and Figure S8C, Supporting Information) while not affecting the hypotonic stress‐induced intracellular calcium release (Figure S7E, Supporting Information), thus pinpointing the calpain pathway as illustrated in Figure 3A.

Immunoblot analysis of cell lysates also showed that the above inhibitors and calcium chelator to be highly effective in preventing vimentin cleavage and dissociation (Figure 4E): blocking the upstream calcium signal with 2‐APB and BAPTA/AM effectively prevented the generation of cleaved bands, whereas for the calpain inhibitor MDL 28170, a weak ≈48 kDa band was observed, indicating substantial, yet incomplete suppression of vimentin degradation. Together, our immunoblot results corroborate, from a global level, our SRM observations of individual cells.

## 3 Discussion

Hypotonic processes are essential both for their key roles in cell physiology and pathology and for their wide use as cell manipulation tools. Through 3D‐STORM SRM, we showed that hypotonic stress induced rapid degradation of the vimentin cytoskeleton, whereas the actin and microtubule cytoskeletal systems were much less affected (Figure 1). Our finding that disruptions to the cell ultrastructure were fully recoverable under normal cell culture conditions (Figure 2) suggests that the use of hypo‐osmotic swelling for intracellular delivery should be acceptable as long as enough recovery time is provided.^4^, ^5^, ^6^ Conversely, for experiments on the use of hypo‐osmotic swelling to modulate membrane tension,^7^, ^8^, ^9^ and protein concentration,^10^, ^11^ it may be necessary to account for the additional intracellular structural changes when interpreting data, as opposed to considering changes in cell geometry alone. Fortunately, in this work we also identified effective, facile means to suppress such structural changes (Figure 4). It would thus be helpful to reexamine if different results might be obtained when the above structural changes are suppressed.

Our results highlight the contrasting responses of different cytoskeletal systems to external stimuli. Unexpectedly, while the actin and tubulin systems are often considered as being more structurally dynamic, we found that under hypotonic stress, fast degradation was specific to the vimentin intermediate filaments (Figure 1). This counterintuitive result, which we later found to be due to a unique IP_3_‐Ca^2+^‐calpain pathway, helps resolve the above‐discussed conundrum of how the otherwise highly stable and rigid vimentin filaments could respond quickly to the hypotonic stress. As the vimentin cytoskeleton plays key roles in defining cell mechanical properties and slowing down intracellular movement,^14^, ^16^, ^18^ the ability to rapidly dissolve the vimentin system provides a potentially helpful mechanism for the cell to reorganize, both morphologically and internally, in response to the hypotonic stress. Given the strong interplays between the vimentin system and the actin and tubulin cytoskeletal systems,^19^, ^20^, ^21^, ^22^ it is further likely that the fast rearrangement of the vimentin cytoskeletal system would next affect the structure and dynamics of the other two.

Our work further demonstrates significant, yet previously overlooked physiological impacts of hypotonic stress‐induced intracellular calcium release, as well as its downstream effects through calpain activation (Figure 3). Notably, we showed that the hypotonic stress‐induced calpain activation could be effectively suppressed by blocking any part of the IP_3_‐Ca^2+^‐calpain pathway (Figure 4). While these results already suggest hypotonic stress could be utilized as a facile, yet potent tool to induce intracellular calcium release and calpain activation, it would be further interesting to ask what other effects may also be triggered by the same pathway, which should also be similarly suppressible by the same inhibitors. Besides intermediate filaments, in neuronal cells and blood cells, members of the spectrin‐based cortical membrane cytoskeleton^25^, ^46^, ^47^ are also important substrates of calpain.^34^, ^48^ Indeed, for the neuron axon initial segment, it has been shown that injury‐induced increase in cytosolic calcium activates calpain toward the rapid degradation of βIV spectrin and ankyrin G, hence an important mechanism of neuronal injury.^49^ Meanwhile, in the ocular lens, Ca^2+^ elevation caused by altered ubiquitin activates calpain toward the degradation of vimentin, fodrin (αII spectrin), and other proteins, hence developmental defects and cataract.^50^ Our finding of hypotonic stress‐induced Ca^2+^ elevation and calpain activation may thus carry broad implications for the structure and function of cells under related physiological and pathological conditions.

## 4 Conclusions

In summary, with 3D‐STORM super‐resolution microscopy, we have unveiled an unexpected, fast, yet reversible dissolution of the vimentin intermediate filament system for cells under hypotonic stress. Counterintuitively, the integrity of the supposedly more dynamic actin and tubulin systems was mostly unaffected under the same conditions. Further integrating results from calcium imaging, biochemical analysis, and inhibitor treatments, we next showed that this vimentin‐specific fast cytoskeletal degradation is due to proteolysis by the calcium‐dependent protease calpain, via a unique IP_3_‐Ca^2+^‐calpain pathway. Together, our findings highlight an unexpected, fast degradation mechanism for the vimentin cytoskeleton in response to the external stimuli, and point to the significant, yet previously overlooked physiological impacts of hypotonic stress‐induced intracellular calcium release on cell ultrastructure and function.

## 5 Experimental Section

*Reagents*: DMEM and Hank's balanced salt solution (HBSS) were from Gibco. EM‐grade paraformaldehyde (15714) and glutaraldehyde (16365) were purchased from Electron Microscopy Sciences (Hatfield, PA, USA). Fura‐2/AM (F0888), MDL 28170 (M6690), 2‐aminoethyl diphenylborinate (2‐APB) (D9754), cysteamine (30070), glucose oxidase (G2133), catalase (C30), 2‐(N‐morpholino)ethanesulfonic acid (MES) (69892), bovine serum albumin (A3059), and other general reagents were obtained from Sigma‐Aldrich (St Louis, MO, USA). BAPTA/AM (B6769) and Alexa Fluor 647‐conjugated phalloidin (A22287) were from Invitrogen (Carlsbad, CA, USA). Primary antibodies used for immunofluorescence: Vimentin, chicken polyclonal, Millipore AB5733; Tubulin, mouse monoclonal, Abcam ab7291. Alexa Fluor 647‐conjugated secondary antibodies were from Invitrogen (A31571 and A21449).

*Cell Culture and Treatments*: COS‐7 cells were cultured in DMEM supplemented with 10% FBS in a humidified CO_2_ incubator with 5% CO_2_ at 37 °C, following standard tissue‐culture protocols. Hypotonic stress was applied by replacing the culture medium with a mixed 1:1 HBSS: water solution [50% HBSS; osmotic pressure was 145 mOsm kg^−1^ measured by a Fiske 210 micro‐osmometer (Norwood, MA, USA)] or pure water at room temperature. For the recovery experiments, the 50% hypo‐osmotic solution or pure water was replaced by the original culture medium, and the sample was kept as above in the incubator for an additional indicated time course. To assess the effects of inhibitors, cells were first preincubated with 75 × 10^−6^ m 2‐APB, 30 × 10^−6^ m MDL 28170 for 15 min in HBSS or 30 × 10^−6^ m BAPTA/AM in HBSS for 30 min, and the sample was treated with pure water containing the same inhibitors at the same concentrations for 5 min.

*Measurement of Cytosolic Ca^2+^ Concentrations ([Ca^2+^]_c_)*: [Ca^2+^]_c_ was measured by a calcium imaging system built on an inverted fluorescent microscope (Olympus IX51, Japan) using the ratiometric Ca^2+^ indicator fura‐2/AM. Cells were first loaded with 5 × 10^−6^ m fura‐2/AM in HBSS for 1 h at room temperature in the dark. After a gentle washing step, cells were bathed in a fresh HBSS (or calcium‐free HBSS, as indicated in the text) solution for [Ca^2+^]_c_ measurement. The cells were alternately excited by a Xenon lamp at 340 and 380 nm using a motorized filter wheel (Lambda 10‐2, Sutter Instrument, Novato, CA, USA). Fluorescence images (filtered at 515 ± 25 nm) were acquired by a charge coupled device (CCD) camera (CoolSNAP fx‐M, Roper Scientific, Tucson, AZ, USA) and analyzed with MetaFluor (Universal Imaging, West Chester, PA, USA). Readout was the ratio of fluorescence intensity excited at 340 nm/excited at 380 nm (F340/F380 ratio, *R*). This readout is converted to Ca^2+^ concentration through in situ calibration using the formula: ${\left[{\mathrm{Ca}}^{2+}\right]}_{c}\;=\;K_{d}\;\left(\frac{R-R_{\min}}{R_{\max}-R}\right)\;\left(\frac{S_{f2}}{S_{b2}}\right)$,where *K*_d_ is the indicator's dissociation constant for Ca^2+^, *R*_max_ and *R*_min_ are *R* measured at saturating Ca^2+^ (substituting the buffer with 5 × 10^−3^ m Ca^2+^ and 10 × 10^−6^ m ionomycin) and zero Ca^2+^ (substituting the buffer with 2 × 10^−3^ m ethylene glycol‐bis(β‐aminoethylether)‐*N*,*N*,*N*′,*N*′‐tetraacetic acid (EGTA) and 10 × 10^−6^ m ionomycin), respectively, and *S_f_*_2_/*S_b_*_2_ is the ratio of fluorescence at 380 nm excitation at zero and saturating Ca^2+^.^51^, ^52^ The values of these parameters for our system were obtained from three independent calibration experiments, yielding *K*_d_ = 220 × 10^−9^ m, *R*_min_ = 0.263 ± 0.034, *R*_max_ = 0.843 ± 0.046, and *S_f_*_2_ */S_b_*_2_ = 2.6 ± 0.2.

*Immunoblot Analysis of Cell Lysates*: Cells were seeded into six‐well plates at 1 × 10^6^ per well and incubated overnight in DMEM with 10% FBS at 37 °C. Total protein lysates were isolated by radio‐immunoprecipitation assay buffer (150 µL per well, R0020, Solarbio, China). The concentration of protein was determined using a BCA assay kit (P0012S, Beyotime, China). Afterward, aliquots (20 µg per lane) were separated by 10% sodium dodecyl sulfate‐polyacrylamide gel electrophoresis and transferred to a nitrocellulose membrane, blocked with 5% BSA at room temperature for 1 h, immunoblotted with rabbit monoclonal antibody against vimentin (1:1000, #5741; Cell Signaling Technology, Danvers, MA, USA) as well as mouse monoclonal antibody against β‐actin (1:20 000, 60008‐1‐Ig; Proteintech, Rosemont, IL, USA) overnight at 4 °C, followed by incubation with antirabbit/mouse horseradish peroxidase‐conjugated secondary antibody (1:1000; A0208/A0216, Beyotime, China) respectively. Finally, an enhanced chemiluminescence detection reagent (B500012, Proteintech, USA) was used for visualization in a Tanon 5200 MultiImage System.

*Cell Fixation and Immunofluorescence*: Cells were seeded on 12 mm glass coverslips in a 24‐well plate at ≈2 × 10^4^ cells per well and cultured for 12 h. For STORM of actin filaments, a previously established fixation protocol was employed:^28^, ^31^, ^32^ The samples were first fixed and extracted for 1 min with 0.3% v/v glutaraldehyde and 0.25% v/v Triton X‐100 in cytoskeleton buffer (CB, 10 × 10^−3^ m MES, pH 6.1, 150 × 10^−3^ m NaCl, 5 × 10^−3^ m EGTA, 5 × 10^−3^ m glucose, and 5 × 10^−3^ m MgCl_2_), and then postfixed for 15 min in 2% (v/v) glutaraldehyde in CB, and reduced with a freshly prepared 0.1% sodium borohydride solution in phosphate buffered saline (PBS). Alexa Fluor 647‐conjugated phalloidin was applied at a concentration of ≈0.4 × 10^−6^ m for 1 h. The sample was briefly washed two to three times with PBS and then immediately mounted for imaging. For imaging of other targets, samples were fixed with 3% w/v paraformaldehyde and 0.1% w/v glutaraldehyde in PBS for 20 min. After reduction with a freshly prepared 0.1% sodium borohydride solution in PBS for 5 min, the samples were permeabilized and blocked in a blocking buffer (3% w/v BSA, 0.5% v/v Triton X‐100 in PBS) for 20 min. Afterward, the cells were incubated with the primary antibody (described above) in blocking buffer for 1 h. After washing in washing buffer (0.2% w/v BSA and 0.1% v/v Triton X‐100 in PBS) for three times, the cells were incubated with the secondary antibody for 1 h at room temperature. Then, the samples were washed three times with washing buffer before mounted for imaging.

*Super‐Resolution Microscopy*: After washing with PBS, the samples were mounted on glass slides with a standard STORM imaging buffer consisting of 5% w/v glucose, 100 × 10^−3^ m cysteamine, 0.8 mg mL^−1^ glucose oxidase, and 40 µg mL^−1^ catalase in Tris‐HCl (pH 7.5).^23^, ^24^ Then, data were collected by 3D‐STORM^23^, ^24^ carried out on a homebuilt setup based on a modified Nikon Eclipse Ti‐E inverted optical microscope using an oil‐immersion objective (Nikon CFI Plan Apochromat λ, 100 ×, numerical aperture = 1.45), as described in our previous work.^53^ Lasers at 405 and 647 nm were introduced into the cell sample through the back focal plane of the objective, and shifted toward the edge of the objective to illuminate ≈1 µm within the glass‐water interface. A strong (≈2 kW cm^−2^) excitation laser of 647 nm photoswitched most of the labeled dye molecules into a dark state, while also exciting fluorescence from the remaining, sparsely distributed emitting dye molecules for single‐molecule localization. A weak (typical range 0–1 W cm^−2^) 405 nm laser was used concurrently with the 647 nm laser to reactivate fluorophores into the emitting state, so that at any given instant, only a small, optically resolvable fraction of fluorophores was in the emitting state. A cylindrical lens was put into the imaging path to introduce astigmatism to encode the depth (*z*) position into the ellipticity of the single‐molecule images.^23^ The Andor iXon Ultra 897 electron‐multiplying CCD recorded images at 110 frames s^−1^ for a frame size of 256 × 256 pixels, and typically recorded ≈50 000 frames for each experiment. 3D‐STORM super‐resolution images were constructed from the single‐molecule images as described previously.^23^, ^24^ For each experimental condition, more than ten cells were imaged, with representative results shown in the main and the Supporting Information figures.

*Live‐Cell Microscopy*: mEos3.2‐Vimentin‐7 was a gift from Michael Davidson (Addgene plasmid # 57485) and transfected into COS‐7 cells using Lipofectamine 3000 (Thermo Fisher). 24 h after transfection, the cells were imaged on an Olympus IX73 inverted wide‐field epifluorescence microscope using an UplanSapo 60 × water‐immersion objective (NA 1.2). A U‐HGLGPS Fluorescence Light Source was used with a green‐emission filter set (Chroma ET470/40x, T495LPXR, and ET525/50m). Images were taken every 30 s using an Andor Zyla 4.2 sCMOS camera.

## Conflict of Interest

The authors declare no conflict of interest.

## Supporting information

Supplementary

Supplementary
