# Supplementary material for: Hypotonic Stress Induces Fast, Reversible Degradation of the Vimentin Cytoskeleton via Intracellular Calcium Release
Source: Adv Sci (Weinh). 2019 Jul 22;6(18):1900865. doi: 10.1002/advs.201900865 (PMC6755523; doi:10.1002/advs.201900865)
Supplement: Supplementary file 1 — Supplementary [file ADVS-6-1900865-s002.pdf]

Copyright WILEY-VCH Verlag GmbH & Co. KGaA, 69469 Weinheim, Germany, 2019.

ADVANCED SCIENCE
Open Access

Supporting Information

for *Adv. Sci.,* DOI: 10.1002/advs.201900865

Hypotonic Stress Induces Fast, Reversible Degradation of the Vimentin Cytoskeleton via Intracellular Calcium Release

*Leiting Pan, Ping Zhang, Fen Hu, Rui Yan, Manni He, Wan Li, Jingjun Xu, and Ke Xu**

Supporting Information

Hypotonic Stress Induces Fast, Reversible Degradation of the Vimentin Cytoskeleton via Intracellular Calcium Release

*Leiting Pan, Ping Zhang, Fen Hu, Rui Yan, Manni He, Wan Li, Jingjun Xu, and Ke Xu**

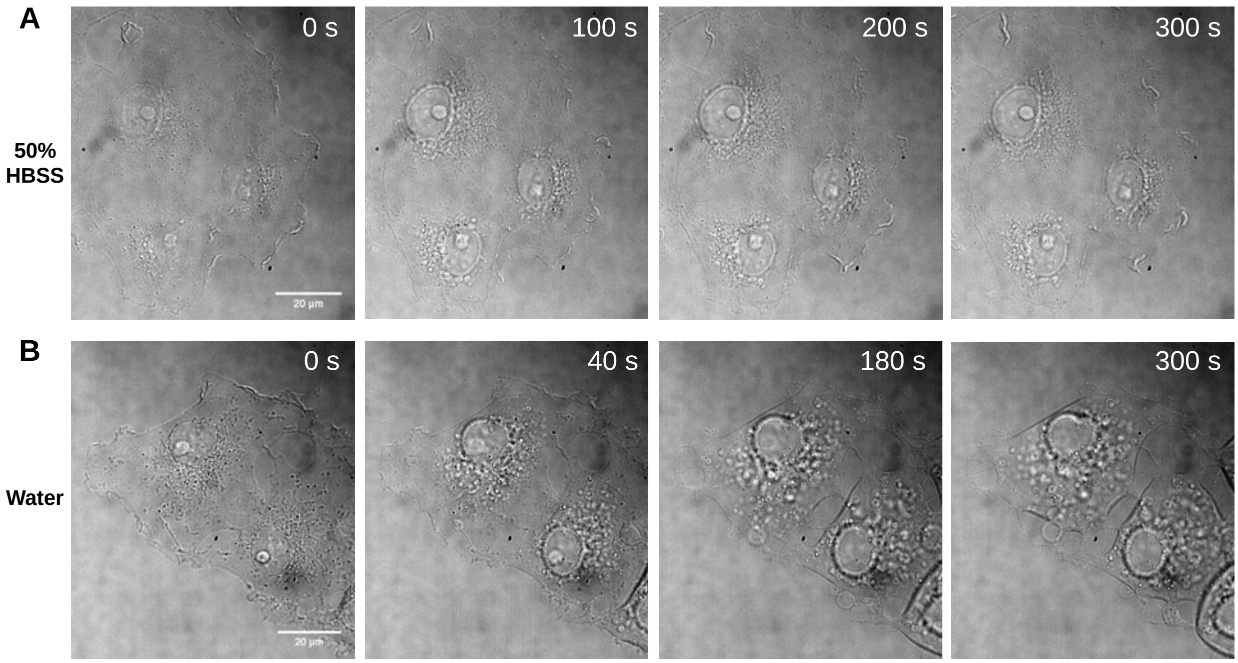

Figure S1. DIC microscopy of cell morphology under hypotonic stress. A) Image sequences for COS-7 cells treated with 50% HBSS. B) Image sequences for COS-7 cells treated with pure water.

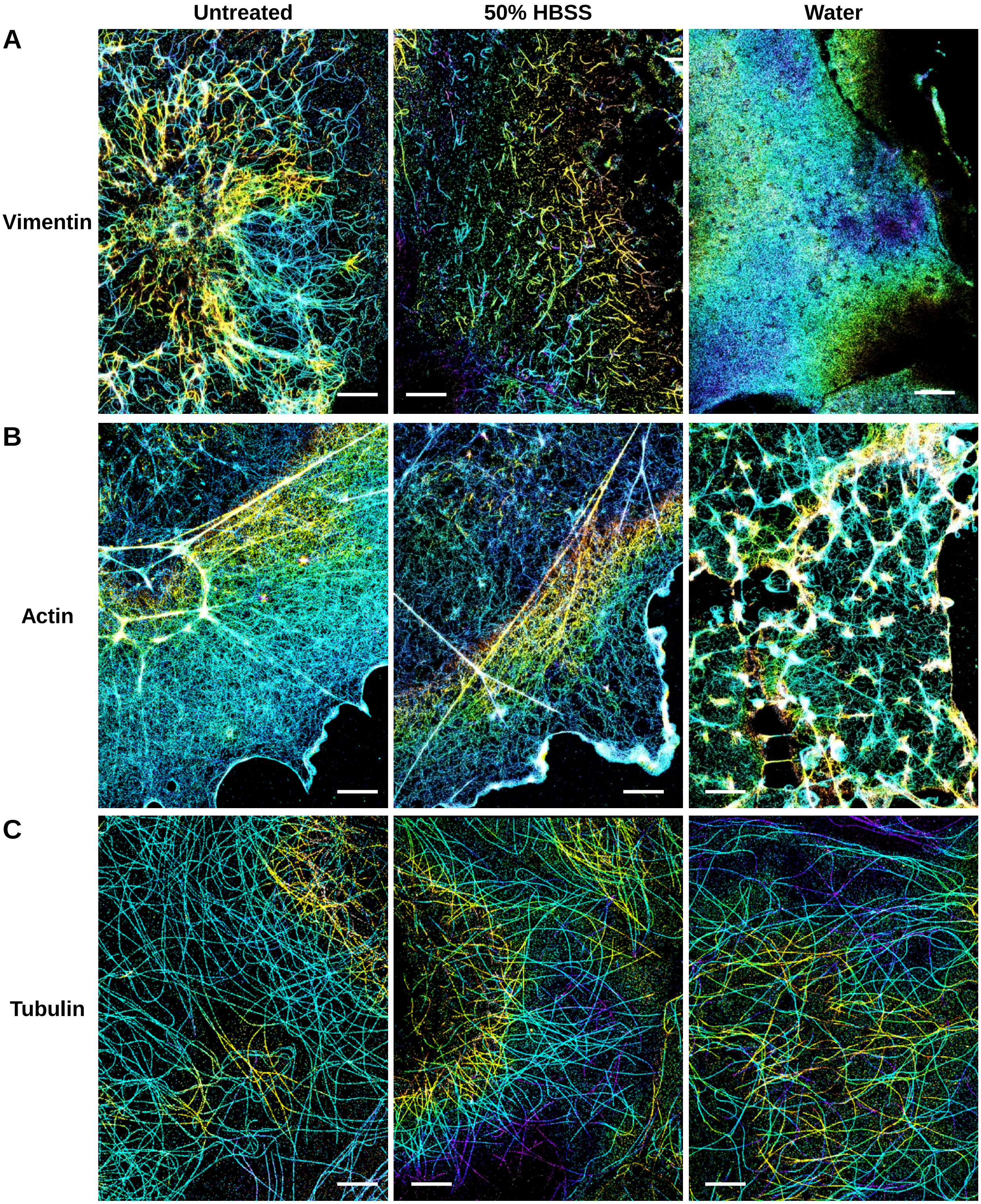

Figure S2. Additional 3D-STORM images of the three cytoskeletal systems under hypotonic stress. A) Immunolabeled vimentin in COS-7 cells untreated (left), treated by 50% HBSS for 5 min (center), and treated by pure water for 5 min (right). B) Phalloidin-labeled F-actin. C) Immunolabeled alpha-tubulin. Scale bars: 4 µm.

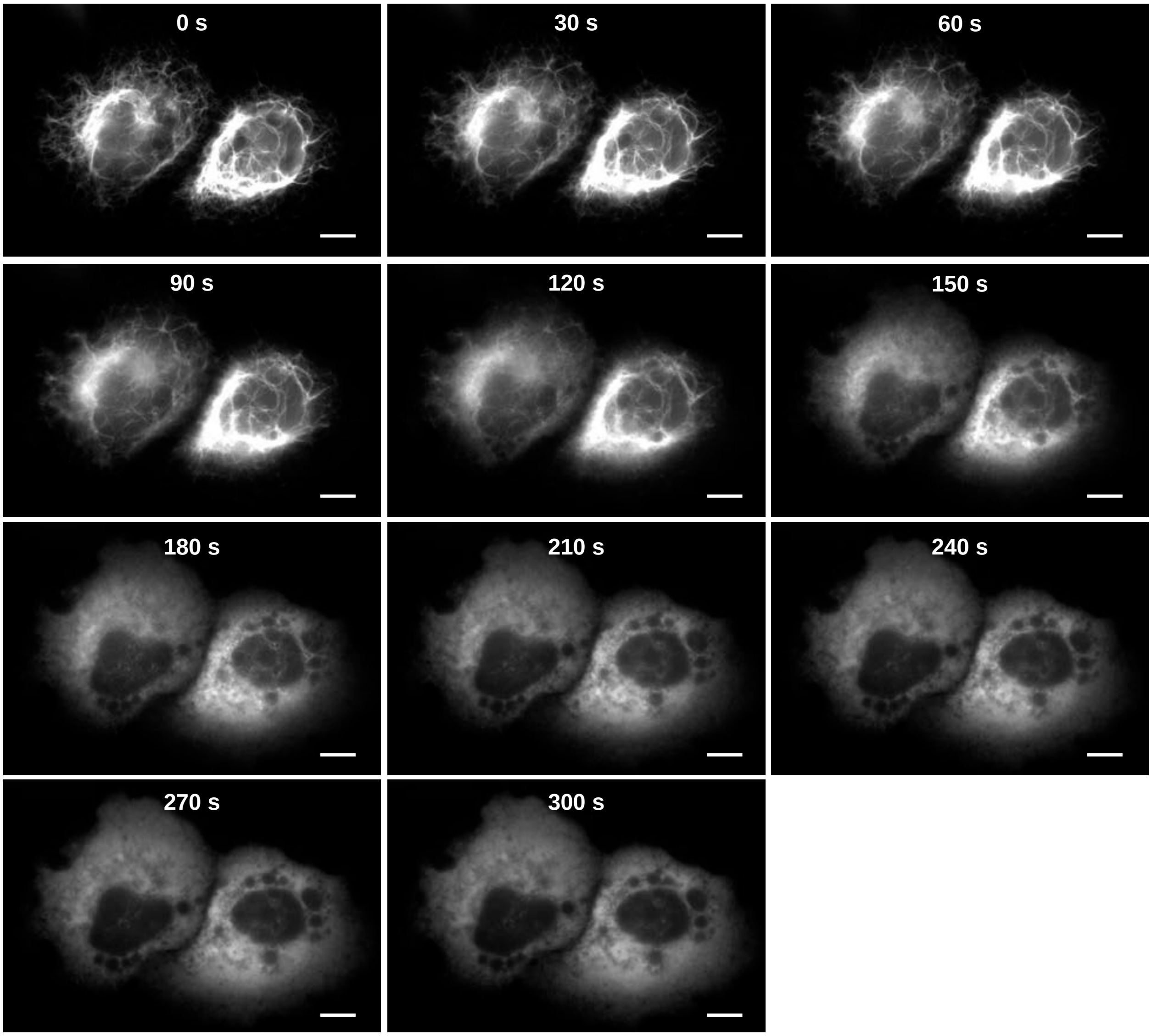

Figure S3. Live-cell microscopy of the vimentin degradation process upon water treatment. Shown: A series of epifluorescence images for two COS-7 cells transfected with mEos3.2-vimentin, at different time points (0-300 s) after treatment with pure water. Scale bars: 10 µm.

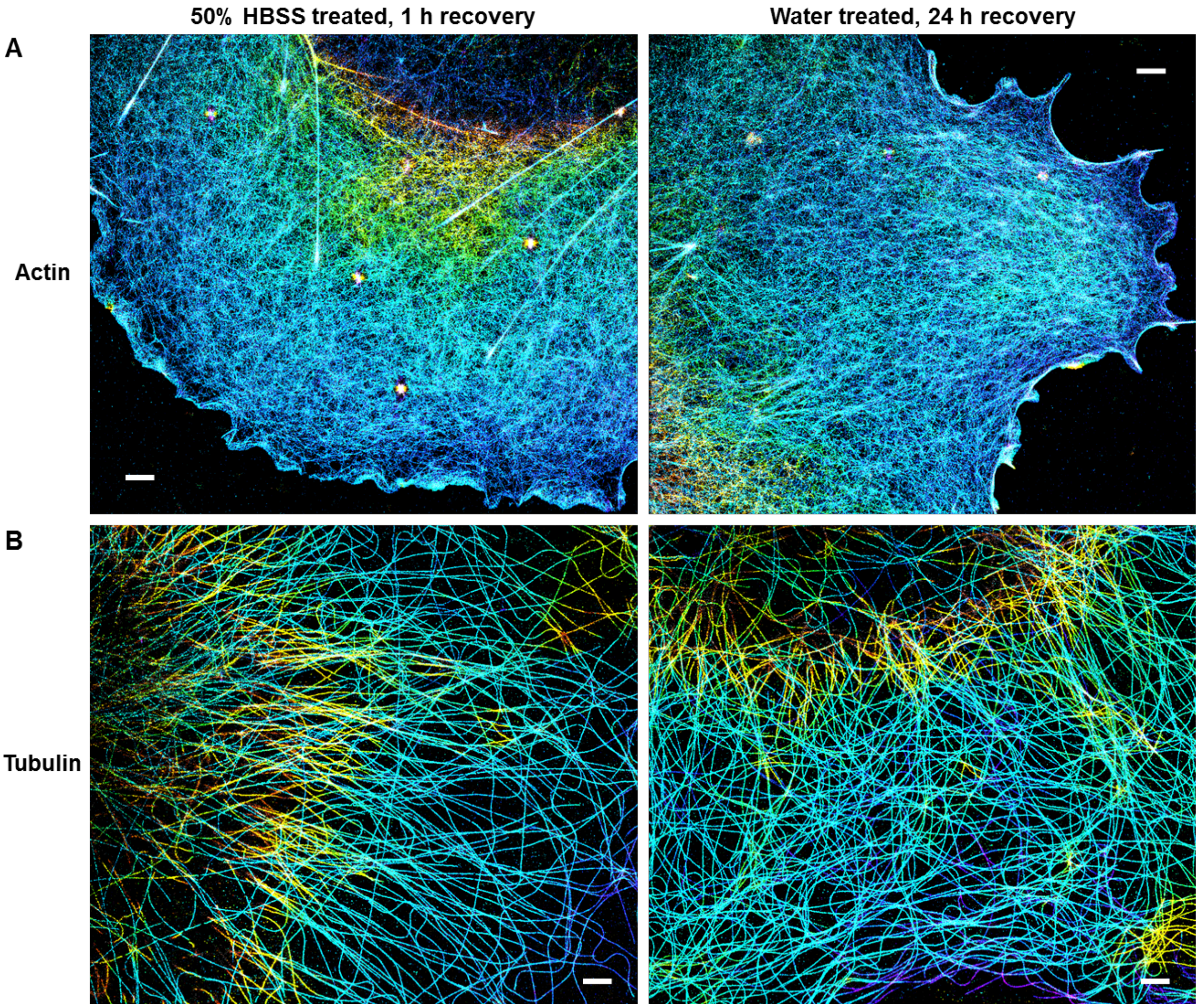

Figure S4. Recovery of the actin and tubulin cytoskeletal systems. A) 3D-STORM images of phalloidin-labeled F-actin in COS-7 cells that had been treated with 50% HBSS for 5 min and then allowed to recover in the regular culture medium for 1 h (left), and that had been treated with pure water for 5 min, and then allowed to recover in the regular culture medium for 24 h (right). B) 3D-STORM images of immunolabeled alpha-tubulin for cells under the same conditions. Scale bars: 2 µm.

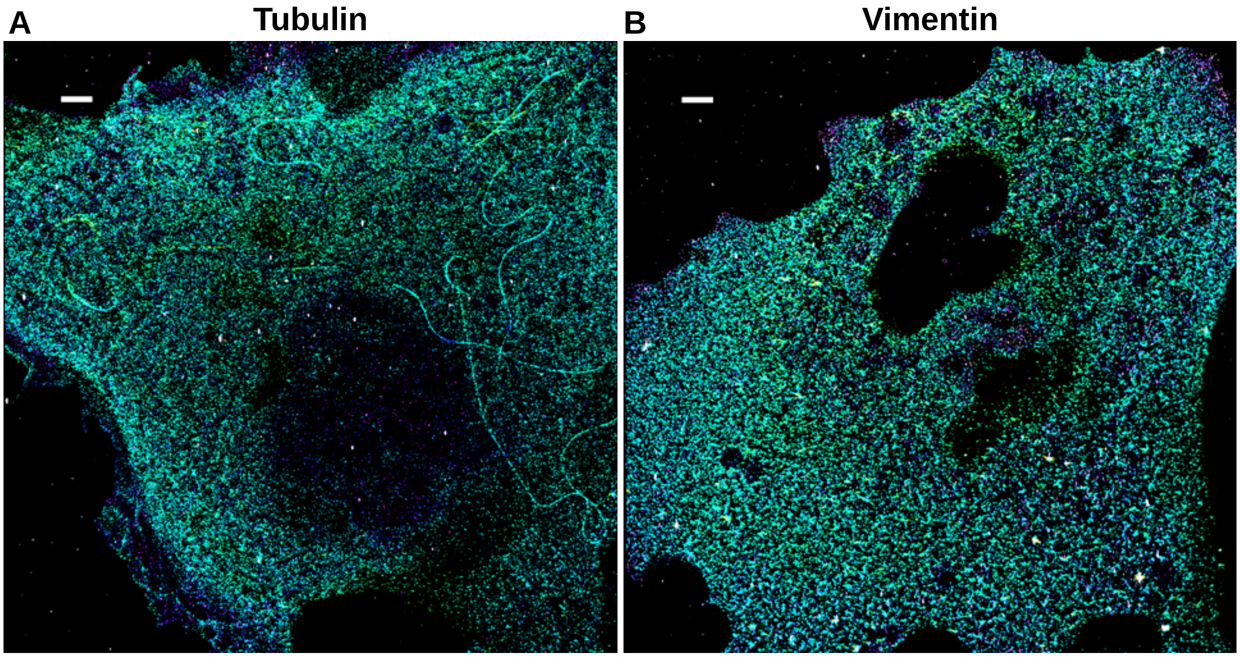

Figure S5. The fast degradation of the vimentin cytoskeleton under hypotonic stress is independent of the microtubule cytoskeletal system. A) 3D-STORM image of immunolabeled alpha-tubulin for a COS-7 cell treated with 10 µM of nocodazole for 1.5 h, showing effective removal of the microtubule cytoskeletal system. B) 3D-STORM image of immunolabeled vimentin in another cell first treated by the same condition (10 µM of nocodazole for 1.5 h), and then treated by pure water for 5 min. Scale bars: 2 µm.

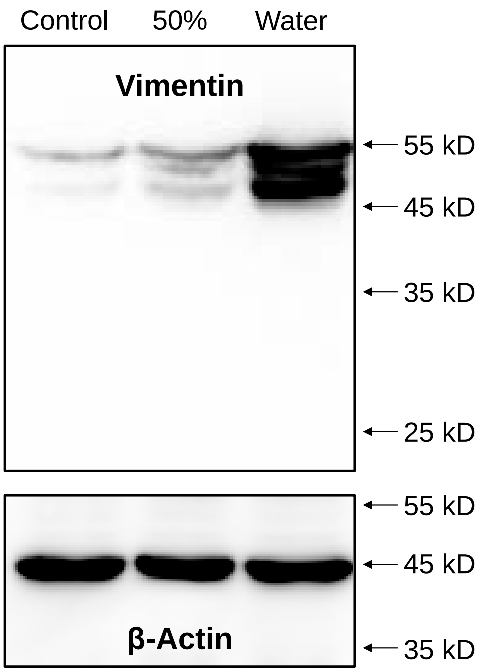

Figure S6. Additional immunoblot results, comparing the lysates of untreated (control) cells, cells treated with 50% HBSS for 5 min, and cells treated with water for 5 min.

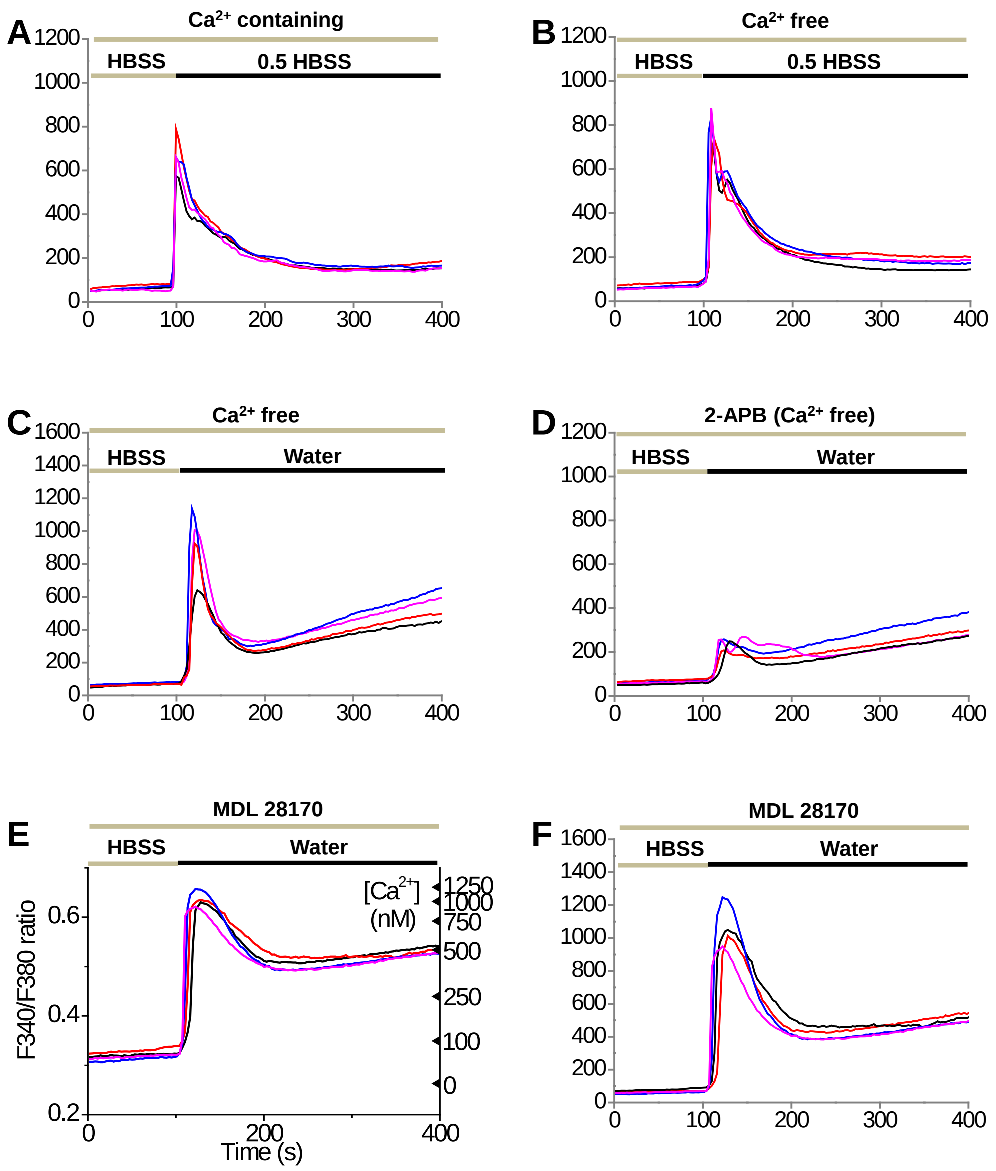

Figure S7. Additional plots of calcium imaging results. A-D) The converted Ca^{2+} concentration plotted on a linear scale for the same results shown in Figure 3C-F in the main text. E,F) The measured calcium signal for cells upon water treatment in the presence of 30 µM MDL 28170, on linear scales of the original readout (E) and the Ca^{2+} concentration (F), respectively.

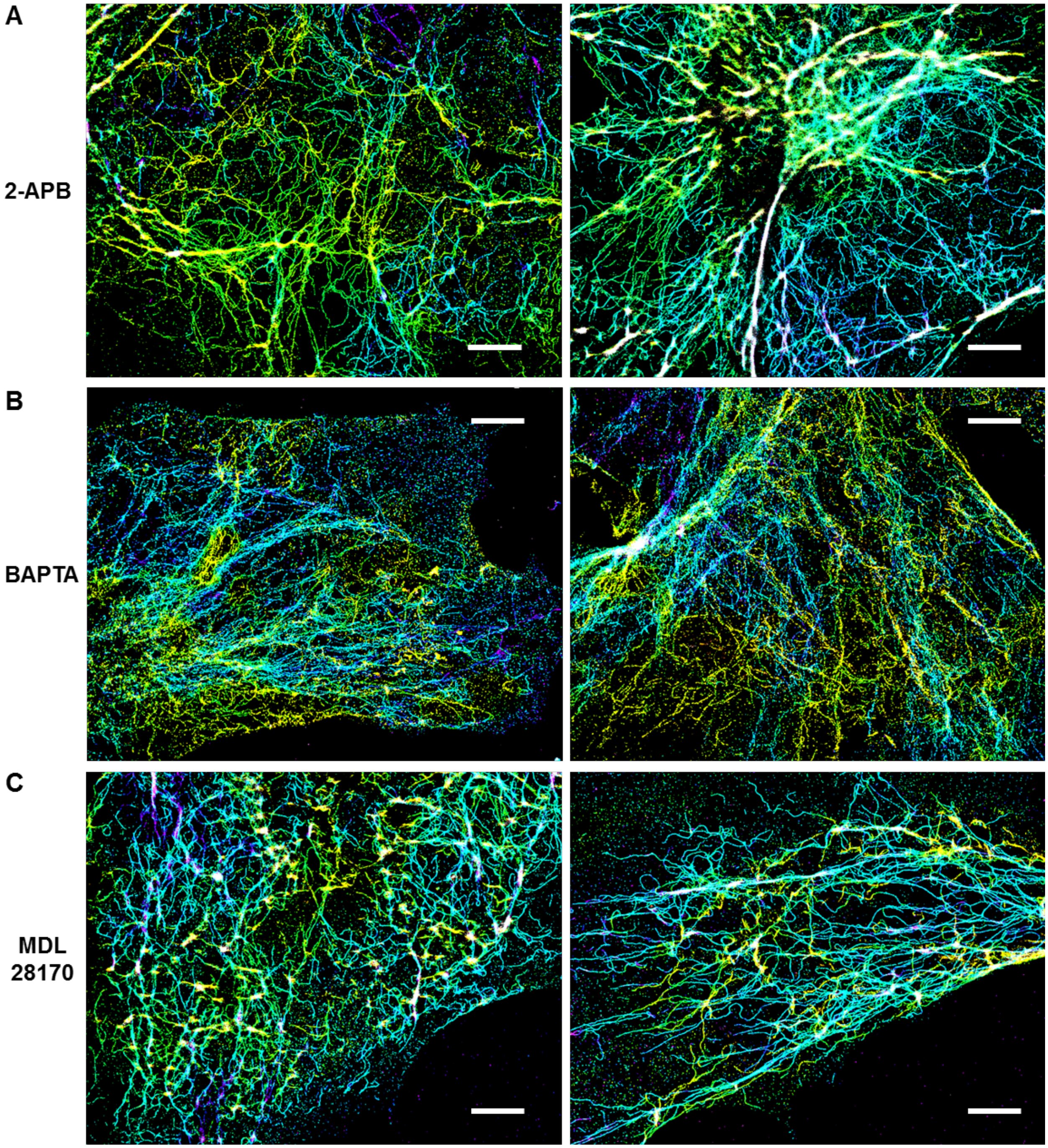

Figure S8. Additional examples of preserving the vimentin cytoskeleton against hypotonic stress through blocking the IP_3-Ca^{2+}-calpain pathway. A) 3D-STORM images of immunolabeled vimentin in COS-7 cells treated with water for 5 min in the presence of 75 μM 2-APB. B) Cells treated with water for 5 min in the presence of 30 μM BAPTA/AM. C) Cells treated with water for 5 min in the presence of 30 μM MDL 28170. Scale bars: 4 μm.

Movie S1. A video clip of the live-cell microscopy data shown in Figure S3.
